# Antipredator responses of three *Daphnia* species within the *D. longispina* species complex to two invertebrate predators

**DOI:** 10.1002/ece3.10841

**Published:** 2024-01-09

**Authors:** Marjohn Yucada Baludo, Pelita Octorina, Andrew Beckerman, Dietmar Straile

**Affiliations:** ^1^ Limnologisches Institut Universität Konstanz Konstanz Germany; ^2^ Department Aquaculture Muhammadiyah University of Sukabumi Sukabumi Indonesia; ^3^ School of Biosciences, Ecology and Evolutionary Biology University of Sheffield Sheffield UK

**Keywords:** antipredator strategies, *Bythotrephes*, *Daphnia*, *Daphnia longispina* species complex, *Leptodora*, morphological defenses

## Abstract

Prey communities in natural environments face a diverse array of predators with distinct hunting techniques. However, most studies have focused only on the interactions between a single prey species and one or more predators and typically only one of many induced defense traits, which limits our understanding of the broader effects of predators on prey communities. In this study, we conducted a common garden experiment using five clones each of three *Daphnia* species (*D. cucullata*, *D. galeata*, and *D. longispina*) from the *D. longispina* species complex to investigate the plasticity of predator‐induced defenses in response to two predators in a community ecology setting. Five clones from each species were subjected to predator kairomones from two closely related invertebrate predators that are common in several European lakes, *Bythotrephes longimanus* or *Leptodora kindtii* for a duration of 10 days, and the morphological traits of body size, head size, spina size, and the presence of spinules on the ventral and dorsal carapace margins were measured. We show that among the species within this species complex there are different antipredator reactions to the invertebrate predators. The induced responses exhibited were species, trait, and predator‐specific. Notably, *D. galeata* and *D. cucullata* developed distinctive helmets as defensive mechanisms, while microdefenses were induced in *D. galeata* and *D. longispina*, but not in *D. cucullata*. This demonstrates that the expression of micro‐ and macrodefenses across species was unrelated, highlighting the possible independent evolution of microstructures as defensive modules in *Daphnia*'s antipredator strategies. This study is the first to document both micro‐ and macrodefensive phenotypic plasticity in three co‐occurring *Daphnia* species within the *D. longispina* species complex. The differences in inducible defenses may have a substantial impact on how these three species cohabit with *Bythotrephes* and *Leptodora*.

## Graphical abstract

This study addresses the gap in predator–prey research by investigating the plasticity of predator‐induced defenses within coexisting *Daphnia* species. Using a common garden experiment, responses to two invertebrate predators were examined, revealing species‐specific and trait‐specific antipredator reactions. Unique defensive adaptations and the independent evolution of microstructures as defensive modules were observed, highlighting the complexity of predator‐induced strategies across species.

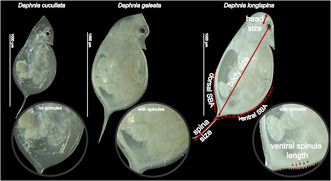

## 1 INTRODUCTION

Predation is a crucial evolutionary force that shapes ecological communities and drives the development of antipredator defenses in many prey species (Tollrian & Harvell, 1999). The evolution of antipredator defenses is determined by several factors. One of these factors is the presence of a reliable cue that indicates the proximity of a threat and activates a defense response in the prey. Additionally, the effectiveness of the prey's defenses against the predator, and the balance between the costs and benefits of developing defenses play crucial roles in this evolutionary process (Tollrian & Harvell, 1999). Prey employ various inducible defenses against their predators, such as morphological changes (Laforsch & Tollrian, 2004; Octorina et al., 2022; Sperfeld et al., 2020), behavioral modifications (Stich & Lampert, 1981), and life‐history traits (Kruppert et al., 2017; Stibor, 1992) to enhance their fitness and reduce predation risk, which are essential for their survival in the environment (Riessen, 2012).

A wide range of organisms, including, for example, protozoans, vascular plants, rotifers, arthropods, and vertebrates have been observed to possess inducible antipredator defenses (Tollrian & Harvell, 1999). In natural environments, prey encounter various predators, each employing distinct hunting and capturing techniques (Laforsch & Tollrian, 2009). Prey can gather and evaluate information about the risk of predation, for example, through chemical cues emitted by predators or their conspecifics (Turner, 2008; Weiss et al., 2012). They can also differentiate between predator species, as seen in *Daphnia pulex* and a freshwater snail *Radix balthica* exhibiting predator‐specific traits when facing multiple predators (Beckerman et al., 2010; Lakowitz et al., 2008; Miner et al., 2005). Nevertheless, most studies examining predator–prey interactions have been limited to investigating interactions between one prey and one predator species (Sperfeld et al., 2020; Tams et al., 2018; Weiss et al., 2015) and single‐induced traits. Even while there has been some research conducted on prey with multiple predators and traits (Diel et al., 2021; Herzog et al., 2016; Laforsch & Tollrian, 2004), there are still surprisingly few studies that concentrate on coexisting prey alongside their complete predator assemblage. This limited scope hinders a comprehensive understanding of the breadth of predator‐induced responses in the prey species that comprise natural ecological communities (Laforsch & Tollrian, 2004; Miner et al., 2005).

The zooplankton genus, *Daphnia* stands out as a well‐studied example of inducible defenses. *Daphnia* exhibits inducible defenses against both vertebrate predators and invertebrate predators (Diel et al., 2020; Tollrian & Harvell, 1999). Most individual studies on the responses of *Daphnia* species have examined their defenses against *Chaoborus* (Beckerman et al., 2010; Carter et al., 2017; Hammill et al., 2008; Laforsch & Tollrian, 2004; Lind et al., 2015; Reger et al., 2018; Sperfeld et al., 2020; Wolinska et al., 2007), *Notonecta* (Diel et al., 2021; Herzog et al., 2016; Ritschar et al., 2020; Weiss et al., 2015), *Triops* (Diel et al., 2021; Herzog et al., 2016; Petrusek et al., 2009; Rabus et al., 2013; Ritschar et al., 2020), and fish (Adamczuk, 2009; Beckerman et al., 2010; Carter et al., 2017; Lind et al., 2015; Reger et al., 2018; Winder et al., 2004; Wojtal‐Frankiewicz et al., 2010).

Despite this huge amount of research done during the last decades (Diel et al., 2020), new types of inducible defenses, particularly morphological defenses, in *Daphnia* continue to be discovered, including the alteration of tiny microstructures (Diel et al., 2021, Ritschar et al., 2020). According to Diel et al. (2021), changes in an organism's microstructure might either be developmentally connected to more obvious changes in its features or represent independent defensive structures to fine‐tune protection against a predator. The first reasoning would suggest that changes at the microstructural level are strongly related to the more significant induced changes that take place during development. For instance, Laforsch and Tollrian (2004) noted that *D. cucullata* displays a strengthened carapace and extended helmet in response to various invertebrate predators, a positive correlation between the macro (helmet) and micro (carapace structure) induced defense. However, in accordance with the “concept of modality” of predators, specialized defenses have generally surpassed general ones throughout evolution. In order to improve the overall defense strategy, subtle changes may thus be important. For example, spinules, micro spike structures along the carapace, might additionally play a role in refining or fine‐tuning the induced phenotype to provide optimal protection against a particular predator with distinctive methods of capture or manipulation (Diel et al., 2021).

In many European lakes, *Daphnia* of the *Daphnia longispina* complex, *D. longispina*, *D. galeata*, and *D. cucullata* co‐occur and dominate the grazing pressure on phytoplankton. In many of these lakes, these *Daphnia* species are exposed to one or two cladoceran predators, *Leptodora kindtii* and *Bythotrephes longimanus*. These two predators differ in predation modes: *Leptodora* is a tactile predator and *Bythotrephes* is a visual predator (Octorina et al., 2022). *Bythotrephes* relies on mechanoreceptors or its large medial compound eye to detect its prey. It then uses its long feeding appendages to grasp the prey and subsequently shred it (Manca et al., 2008). In contrast, *Leptodora* employs a strike tactic for capturing prey and requires direct contact with the prey before initiating an attack (Browman et al., 1989; Manca et al., 2008) and uses a “trap basket” for capturing prey (Branstrator, 2005). Unlike *Leptodora*, *Bythotrephes* are likely capable of successfully feeding on larger prey items because they are not constrained by a feeding basket (Manca et al., 2008). Previous studies have shown that *L. kindtii* induces significantly longer helmets and tail spines on *D. cucullata* (Laforsch & Tollrian, 2004), while *B. longimanus* induces a typical helmet on *D. galeata* (Octorina et al., 2022), but the responses of *D. longispina* to both predators have not been explored.

Here, we investigate the predator‐induced defenses in a community ecology context, evaluating responses among three species within the *D. longispina* species complex which co‐occur with the two invertebrate predators in Lake Constance. We examine plasticity in head, respectively helmet, body, and spina size, as well as in proposed microstructures, the extension of the ventral and dorsal spinule areas, and the length of the ventral spinule (Diel et al., 2021) in response to the two predators.

Both *D. galeata* and *D. longispina* are morphologically similar and adult individuals of these species typically range in body size from 1.2 to 2.5 mm. As *D. cucullata* is smaller (Ogorelec et al., 2022; Stich et al., 2005) compared with the other two species, we expect that small *D. cucullata* might be more vulnerable to invertebrate predation. Besides size, the species also differ in antipredator behavior. *D. longispina* performs diel vertical migration (DVM) (Geller, 1986; Stich & Lampert, 1981), while *D. cucullata* and *D. galeata* stay in the upper water strata (0–20 m) (Geller, 1986; Ogorelec et al., 2022; Stich & Lampert, 1981). As the two invertebrate predators also inhabit the upper water layers (Stich, 1989), *D. galeata* and *D. cucullata* (not performing DVM), but not *D. longispina* (performing DVM) are exposed to the two predators throughout a 24‐h day.

Given this background and the potential for co‐variation between macro and micro induced defense traits, we formulated and tested four hypotheses: (1) based on the differences in predation behavior between the two predator species, we anticipate distinct responses in the daphnids; (2) we expect variations in the expression of defensive traits among the *Daphnia* species, with the most vulnerable *D. cucullata* showing the strongest responses, while the least vulnerable *D. longispina* displaying only weak responses to predator kairomones; (3) given the “connectedness sensu Diel et al. (2021)” between small‐scale defensive qualities and the large‐scale defensive traits against invertebrate predators, we expect that all three species will display microdefenses in response to both predators; (4) finally, we expect that the expression of microdefenses would be directly related to the expression of macrodefenses, emphasizing the complexity of the defensive strategies.

## 2 MATERIALS AND METHODS

### 2.1 Origin of *Daphnia* clones

*Daphnia* clones were collected in Upper Lake Constance, located in Central Europe and bordering Germany, Switzerland, and Austria, which is the main basin of the large peri‐alpine Lake Constance (Güde & Straile, 2016). The number of *Daphnia* species has changed there during the last century. While until the mid‐20th century only *D. longispina* was found in the lake, *D. galeata* was able to invade the lake with eutrophication in the 1950s (Straile, 2015). More recently, *D. cucullata* established large densities (Ogorelec et al., 2022) and currently all three species co‐occur in the lake. Both predators, *B. longimanus* and *L. kindtii*, have been regularly found in the lake since more than one century (Straile & Geller, 1998). Clones of *Daphnia galeata* were hatched from ephippia, which were collected from the upper 20 cm of a sediment core taken from the lake. In contrast, *Daphnia longispina* and *Daphnia cucullata clones* were collected in Lake Constance using a plankton net (mesh size 140 μm) drawn from 40 to 0 m. The lineages established from these individuals of *D. cucullata* and *D. longispina* are thus isolates, and it is not 100% certain that they represent different clones. However, given the large clonal diversity of *Daphnia* spp. in Lake Constance (Beninde, 2021), and our low numbers of isolates obtained from 100's sampled, we consider it unlikely that two randomly picked individuals from several hundred individuals in one sample share their clonal identity. Consequently, in this paper, we referred to them as clones, acknowledging the potential that two isolates may share the same clonal identity. The three species were cultured in the laboratory for several months before the experiment. The third clutch from the third generation of maternal lines was collected as study organisms. In order to reduce the influence of extraneous variables, all stock cultures of clones were kept under the same conditions (e.g., feeding volume, feeding schedules, temperature, light). Maternal cultures were kept in 1 L glass jars with 800 mL lake water in 20°C (range: 19–21°C) with a 16:8 L:D photoperiod cycle. These cultures were fed with 2 mg C/L green alga *Tetradesmus obliquus* (Culture Collection of Algae, University of Göttingen, Germany, SAG 276‐3a) three times per week and were transferred to a new medium until they reached the third generation.

Lake water was prepared for both *Daphnia* culturing and the experiment by undergoing a process involving filtration through a 0.2‐μm mesh sieve. Subsequently, the filtered water was aerated and allowed to age for 24 h to prevent expression of morphological defenses in *Daphnia* cultures.

### 2.2 Experimental setup and measurements of morphological traits

Since our focus was on studying species differences rather than clonal variations within species, we conducted replications at the species level, with five clones each, rather than at the clonal level. The five clones of each *Daphnia* species, *D. cucullata*, *D. galeata*, and *D. longispina* were placed in three different treatments: *Bythotrephes* (B), Control (C), and *Leptodora* (L). For each clone and treatment, we added three to four 1‐day old *Daphnia* individuals to 200‐mL glass jars containing 150 mL lake water. The number of experimental animals at the start of the experiment was 3 species × 5 clones (5 jars) × 3 treatments × 3–4 daphnids per jar, in total of 45 jars and 164 individuals. Unfortunately, 63 daphnids died during the experiment resulting in 101 daphnids used for morphological measurements. Mortality was mostly caused by the transfer of daphniids into new jars every second day and was highest for fragile *D*. cucullata (65% of all mortality). This caused the loss of two clones of *D. cucullata* in the *Leptodora* (L) treatment. The analysis of spina length variability was conducted using only daphnids with intact spines (*n* = 96). This exclusion involved the removal of two clones of *D. cucullata* in the *Bythotrephes* (B) treatment due to broken spina. Hence, the number of independent experimental units was 43 (3 species × 5 clones × 3 treatments—2) for analyses of body and head sizes and microstructures, and 41 for the analysis of spina size.

Five predators were added to each jar in the predator treatments, which were collected from the lake using a plankton net towed behind a boat. These predators were placed in a cylindrical plastic cage (4 cm diameter and 6 cm height) with a 140‐μm nylon mesh (Octorina et al., 2022) and were not fed with *Daphnia*. In the control treatment, the plastic cage was left empty. Every second day, daphnids were transferred to new jars filled with lake water with *T. obliquus* and freshly collected predators. Throughout the experiment, daphnids were fed with 1 mg C/L of the green alga *T. obliquus* for the first 7 days and 2 mg C/L for the remaining 3 days to ensure also that large and matured daphnids have unlimited food. The experiment was conducted at a constant temperature of 20°C (range: 19–21°C) and a 16:8 L: D photoperiod cycle.

**FIGURE 1:**
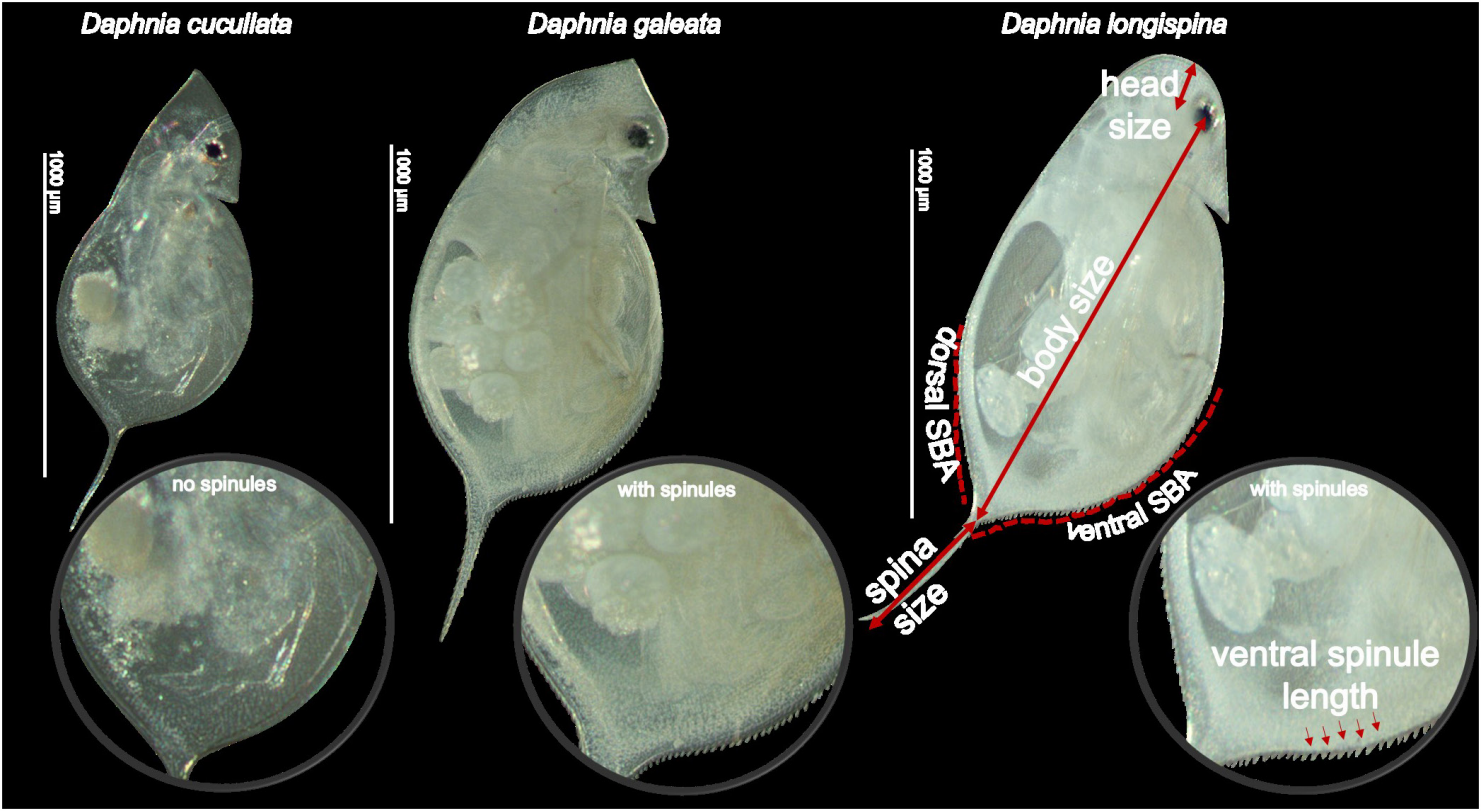
Measurements of various morphological traits of *Daphnia* species and the presence/absence of dorsal and ventral spinules.

At an age of 10 days, the daphnids were preserved in 70% ethanol following shock treatment in 95% ethanol (Black & Dodson, 2003). We chose age 10 for all three species because this age is sufficient to guarantee that all individuals were mature in all treatments. Subsequently, morphological defenses of *Daphnia* individuals were measured in R version 4.2.2 (R Core Team, 2021) with a self‐written script using photographs of the daphnids taken with a Bresser MikroCamII 12 MPu mounted to a Stemi 2000‐C stereomicroscope (Carl Zeiss Werk, Göttingen, Germany). For each *Daphnia*, we measured body size, head size, spina size, and the spinules on the ventral and dorsal carapace margins (Figure 1). Body size was determined from the base of the spina to the mid‐eye, while spina size was measured from the end of the tail spine to the base of the spina. Head size was measured in parallel to the body axis from the top of the head or top of the helmet until the line perpendicular to the body axis crossing the midpoint of the eye. All *Daphnia* individuals were examined for the presence of spinules in the dorsal and ventral carapace margins and for the presence of neckteeth. The ventral spinules bearing area (ventral SBA) was measured from the first ventral spinule to the base of the tail spine, while the dorsal spinules bearing area (dorsal SBA) was measured from the first dorsal spinule to the base of the tail spine. Additionally, the length of the ventral spinules was measured from the base of the spinule to its tip, with five spinules per animal measured (Diel et al., 2021). Unfortunately, one *D. longispina* and eight *D. galeata* pictures were not suitable to measure the microstructures. This reduced the number of observations, but not the number of independent experimental units in our dataset regarding microdefenses.

### 2.3 Test of hypotheses 1 and 2: Predator‐specific responses and different level of defensive trait expressions in *Daphnia*

Variations in body size, head size, spina size, dorsal SBA, ventral SBA, and ventral spinule length were then analyzed using linear mixed‐effect models (Kuznetsova et al., 2017) with body size, treatment, and species as fixed effects and clonal identity as a random effect. This accounts for non‐independence of the daphnids from each clone which were kept in one jar. Body size was centered in the model to eliminate co‐variation between body size and *Daphnia* species identity. Hence, centered body size in the models predicts within‐species trait variability, whereas between‐species trait variability is predicted by species identity. We used likelihood ratio tests (ANOVA and RANOVA functions) (Kuznetsova et al., 2017) to evaluate the significance of fixed and random effects. If significant treatment effects were found, we conducted pairwise post hoc comparisons using the Tukey method (Lenth et al., 2023).

### 2.4 Test of hypothesis 3: Microdefenses in all three species

To test the predictions regarding the occurrence of microdefenses in all three species, we calculated the mean trait residual for dorsal SBA, ventral SBA, and ventral spinule length, and compared these values among the *Daphnia* species. The linear mixed‐effect model for dorsal SBA, ventral SBA, and ventral spinule length was then utilized to analyze treatment and species effects, followed by post hoc comparisons.

### 2.5 Test of hypothesis 4: Co‐variation of macrodefenses and microdefenses

Finally, to examine co‐variation between macrodefenses and microdefenses, we first calculated the residuals of regressions between each trait and body size separately for each species. Next, we calculated the mean residual values for each trait and clone and determined the correlation for each combination of pairwise traits and across *Daphnia* species. This allowed us to assess the presence of trade‐offs versus positive co‐variation between the different traits. For multiple comparisons, we corrected the *p*‐values using a Benjamini–Hochberg adjustment (Benjamini & Hochberg, 1995).

Statistical analyses were conducted using R version 4.2.2 (R Core Team, 2021). All models were constructed using lme4 (Bates et al., 2015) and analyzed using lmerTest (Kuznetsova et al., 2017). Post hoc testing, where appropriate, was carried out using the emmeans package (Lenth et al., 2023). Figures were generated using the ggplot2 package (Wickham, 2016) and the pairs R function.

## 3 RESULTS

### 3.1 Hypotheses 1 and 2: Predator‐specific responses and the differences in the defensive trait expressions in *Daphnia*

**FIGURE 2:**
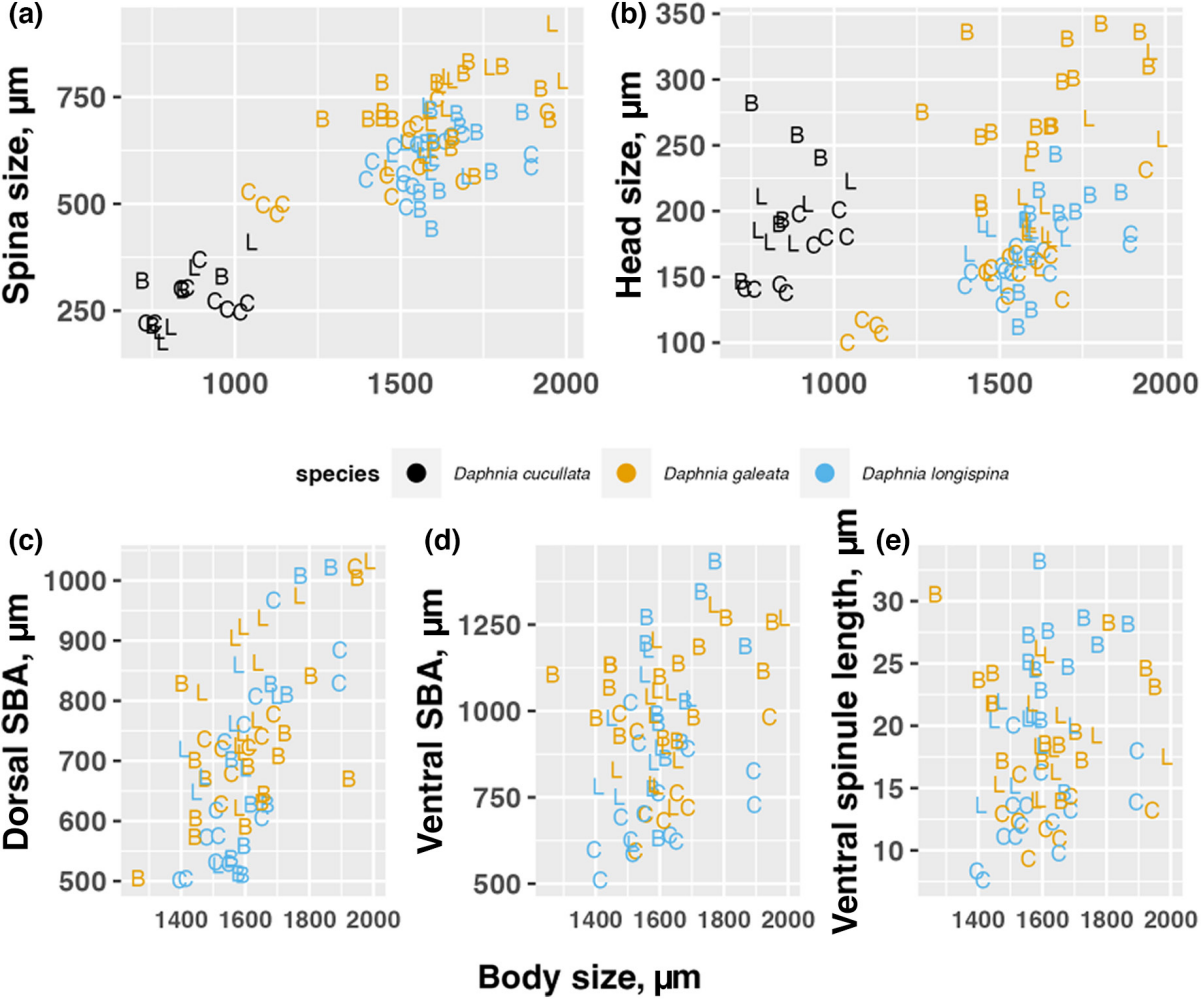
Relationship between body size and spina size, head size, dorsal SBA, ventral SBA, and ventral spinule length for the three *Daphnia* species. Each treatment condition is shown with distinct letters (*Bythotrephes* (B), Control (C), and *Leptodora* (L)).

**FIGURE 3:**
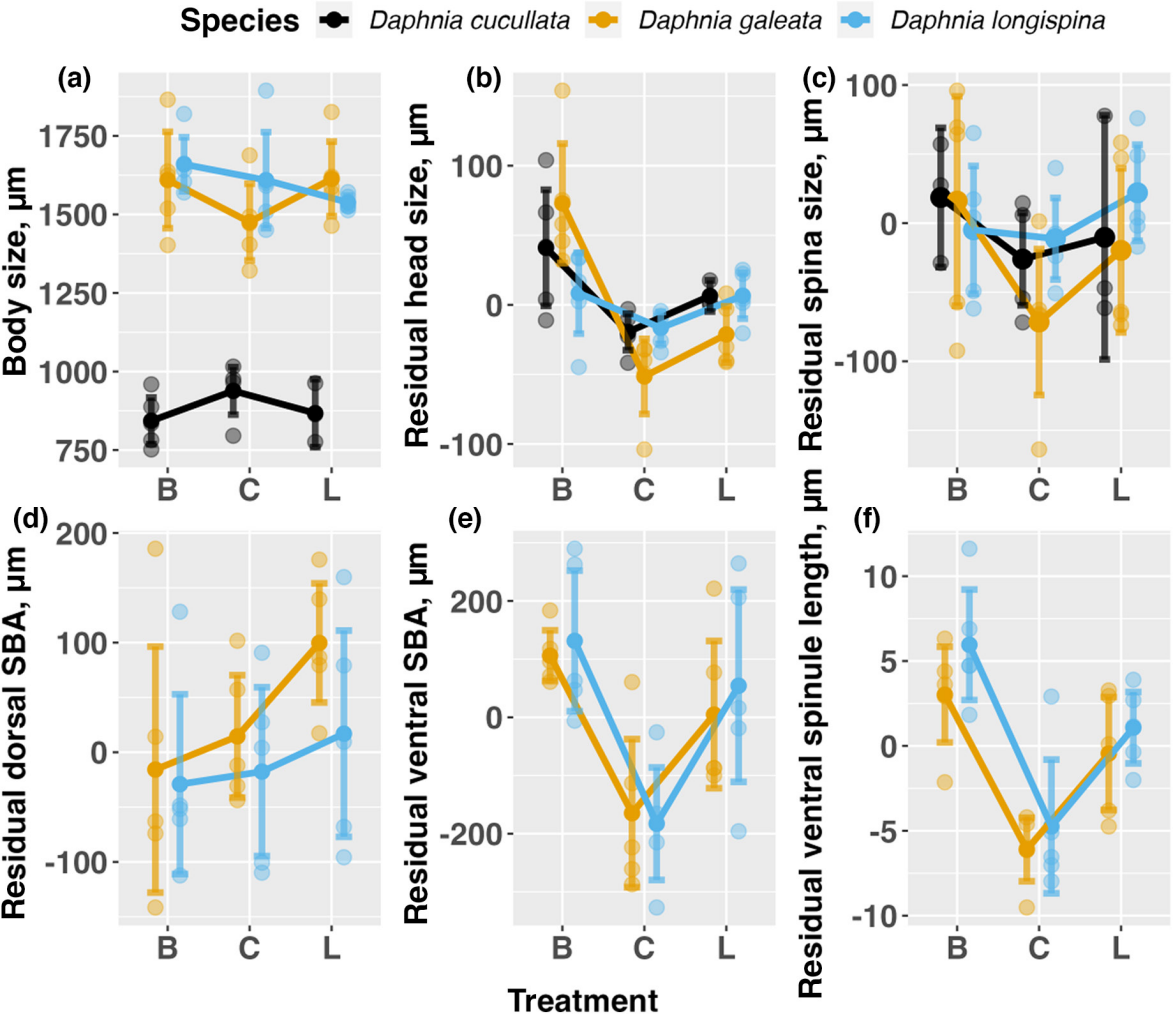
Responses (means ± 2 standard errors) of the three *Daphnia* species to three treatments (B: *Bythotrephes*, C: Control, L: *Leptodora*) for the traits body size, residual head size, residual spina size, residual dorsal SBA, ventral SBA, and residual ventral spinule length. Small dots represent the clonal means for the different treatment—species combinations.

After a growth period of 10 days, it was evident that the body size of *D. cucullata* was significantly smaller, mostly below 1000 μm, compared to the other two species, which generally reached body sizes between 1500 and 2000 μm (Figures 2 and 3a). The presence of predators led to an increase in the body size of *D. galeata* (B – C: *t*_84.7_ = 2.769, *p* = .0187; C – L: *t*_84.2_ = −3.355, *p* = .0034) (Figures 2 and 3a), while the effects of predator kairomones on the body size of the other two species were not as clear (Table 1a, significant treatment × species interaction).

**TABLE 1:** Summary of the linear mixed‐effects model on the morphological trait responses of *Daphnia* community against two co‐occurring invertebrate predators.

Traits	Fixed effects							Random effect
	Body size	Tr	Species	Body size: Tr	Body size: Species	Tr: Species	Body size: Tr: Species	1 | clone
a. Body size		*F*_2,86.0_ = 0.901ns	*F*_2,13.0_ = 91.1***			*F*_4,85.8_ = 3.33*		LRT_1_ = 4.91*
b. Head size	*F*_1,81.9_ = 18.6***	*F*_2,75.9_ = 50.6***	*F*_2,10.9_ = 5.81*	*F*_2,77.7_ = 2.47ns	*F*_2,83.7_ = 0.134ns	*F*_4,75.5_ = 16.3***	*F*_4,75.6_ = 1.59ns	LRT_1_ = 13.7***
c. Spina size	*F*_1,65.6_ = 15.9***	*F*_2,74.7_ = 2.52ns	*F*_2,12.6_ = 82.2***	*F*_2,78.4_ = 1.21ns	*F*_2,78.7_ = 1.07ns	*F*_4,74.2_ = 2.65*	*F*_4,75.0_ = 2.90*	LRT_1_ = 16.1***
d. Dorsal SBA	*F*_1,53.4_ = 55.8***	*F*_2,55.3_ = 7.17**	*F*_1,6.64_ = 8.76*	*F*_2,53.9_ = 2.13ns	*F*_1,53.4_ = 2.45ns	*F*_2,55.3_ = 0.246ns	*F*_2,53.9_ = 5.04**	LRT_1_ = 2.33ns
e. Ventral SBA	*F*_1,54.8_ = 6.37*	*F*_2,56.1_ = 18.2***	*F*_1,8.47_ = 1.28ns	*F*_2,55.1_ = 1.72ns	*F*_1,54.8_ = 0.81ns	*F*_2,56.1_ = 0.347ns	*F*_2,55.1_ = 0.830ns	LRT_1_ = 3.43ns
f. Ventral spinule length	*F*_1,49.6_ = 0.522ns	*F*_2,56.9_ = 38.6***	*F*_1,8.66_ = 1.93ns	*F*_2,56.1_ = 0.17 ns	*F*_1,49.6_ = 1.89ns	*F*_2,56.9_ = 1.21ns	*F*_2,56.1_ = 0.102ns	LRT_1_ = 1.05ns

*Note*: The analysis included fixed effects such as centered body size, head size, spine size, dorsal spinule bearing area (SBA), ventral SBA, and ventral spinule length using type III analysis of variance with Satterthwaite's method to examine the significance of these fixed effects. Clonal identity was treated as a random effect, and Likelihood ratio tests were employed to evaluate its impact on the model. “Tr” refers to the three treatments: *Bythotrephes*, Control, *Leptodora*. The model employed an equal count of observations (*n* = 101) for body size and head size, whereas the count differed for spina size (*n* = 96) because data points were excluded for daphnids with broken spines in the *Bythotrephes* treatment. Additionally, the observation count varied for the microdefenses model (*n* = 71) due to the absence of these defenses in *D. cucullata*. Significance levels are given as: ns, not significant; *p < .05; **p < .01; ***p < .001.

We did not observe any neckteeth for any *Daphnia*. All other traits were influenced by predator kairomones in a species‐specific manner, and except for the ventral spinule length, all trait lengths were related to body length (Figures 2 and 3). This relationship held even when body lengths were centered for each species (Table 1). Regarding body size, head size, and spina size, significant clonal variability was found, indicated by significant random intercepts (Table 1).

The kairomones of both predators increased head sizes due to the development of a helmet in *D. cucullata* (B – C: *t*_77.8_ = 4.222, *p* = .0002; C – L: *t*_78.5_ = −2.808, *p* = .0171) and *D. galeata* (B – C: *t*_72.6_ = 13.204, *p* < .0001; C – L: *t*_72.1_ = −3.224, *p* = .0053). However, this effect was not observed in *D. longispina* (Figures 2 and 3b, Table 1b, significant species x treatment interaction). Notably, the increase in *D. galeata* head sizes was greater in the *Bythotrephes* treatment compared with the *Leptodora* treatment (*t*_71.5_ = 9.637, *p* < .0001), but no significant difference was detected in *D. cucullata* between the two predator treatments (*t*_76.2_ = 1.455, *p* = .3181).

The influence of kairomones on spina length depended on body size and *Daphnia* species (significant three‐way interaction). This was mainly due to the response of *D. galeata* in the *Leptodora* treatment, where the spina length–body size slope was steeper than in the *Bythotrephes* and control daphnids, resulting in large spines only for large daphnids (Figure 2a). The spina length–body size slopes were comparable between the control and *Bythotrephes* treatments for *D. galeata* and larger spines were observed in the *Bythotrephes* treatment compared with the control treatment at similar body sizes (*t*_68.5_ = 3.939, *p* = .0006).

### 3.2 Hypothesis 3: Microdefenses in all three species

We found qualitative differences between the three species regarding microdefenses: *D. cucullata* did not develop microstructures in the presence of predators in contrast to the other two species. Consequently, we used only the data for *D. galeata* and *D. longispina* in the mixed models for microstructures. The effect of kairomones on dorsal SBA depended on body size and *Daphnia* species (significant three‐way interaction). This threefold interaction was mostly caused by the *Bythotrephes* treatment response, where dorsal SBA for *D. longispina* increased more steeply with body size than SBA of *D. galeata* (Figure 2c). Ventral SBA and ventral spinulae lengths did not differ between the two species, but were larger in both kairomones treatments compared to the control (Figure 3e: *D. galeata* (B—C: *t*_53.7_ = 4.118, *p* = .0004) and *D. longispina* (B—C: *t*_57.8_ = 4.541, *p* = .0001; C—L: *t*_53.9_ = −3.023, *p* = .0105), Figure 3f: *D. galeata* (B—C: *t*_54.1_ = 5.261, *p* < .0001; C—L: *t*_52.5_ = −3.957, *p* = .0007) and *D. longispina* (B—C: *t*_58.6_ = 7.198, *p* < .0001; C—L: *t*_55.0_ = −3.217, *p* = .0061)).

### 3.3 Hypothesis 4: Co‐variation of macrodefenses and microdefenses

**FIGURE 4:**
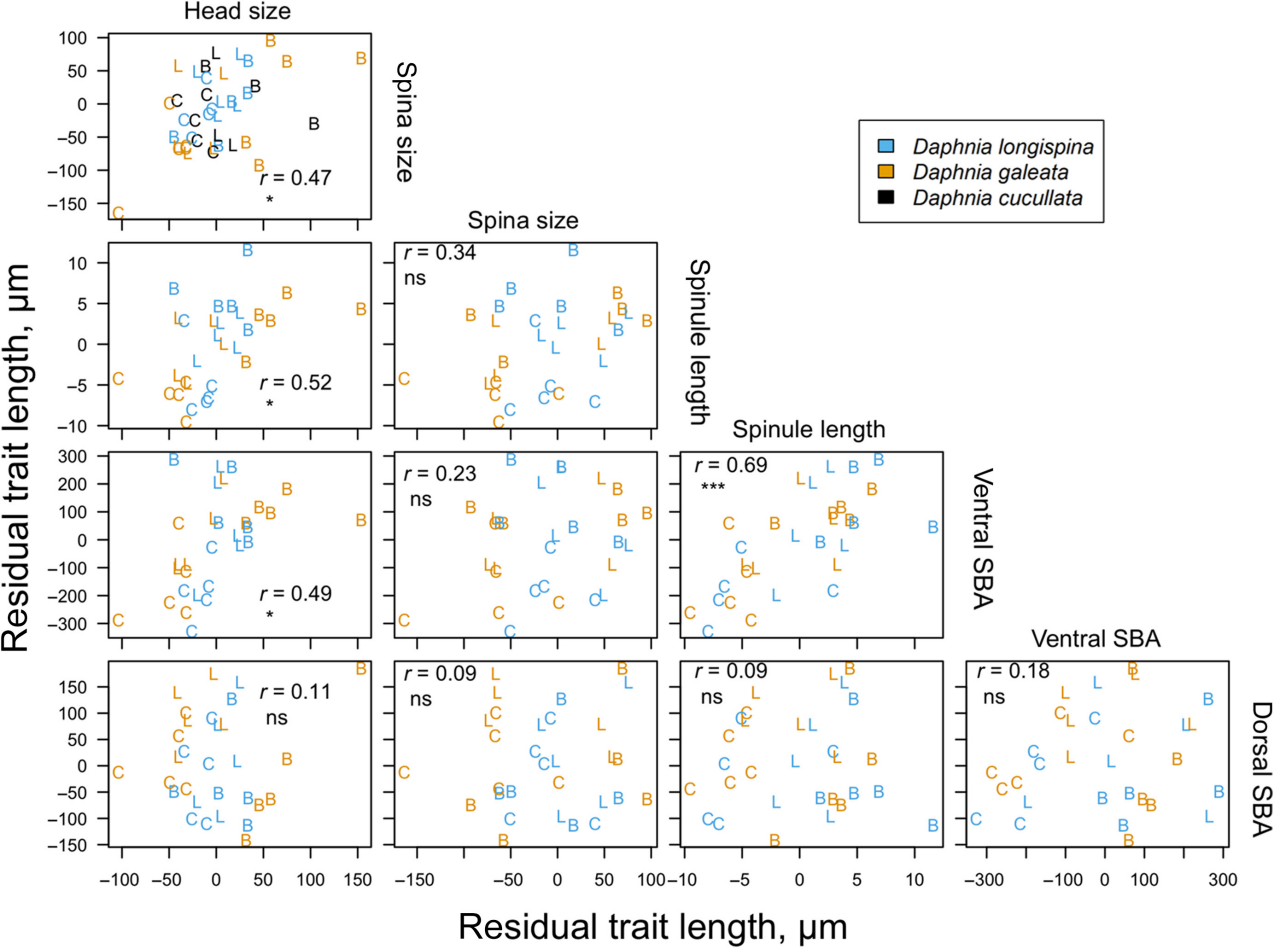
Relationships among various residual mean traits. The colored letters in the figure correspond to three treatments, *Bythotrephes* (B), Control (C), and *Leptodora* (L) showing the mean of the clone among three *Daphnia* species. The *r* values in each box in the figure displays the Pearson correlation coefficient for the respective pair of residual traits, and asterisks indicate significant co‐variation between the respective traits (*p < .05, **p < .01, ***p < .001 (Ross, 2017)).

Morphological traits adjusted for body size (Figure 4) co‐varied across species and treatments in 4 out of 10 trait combinations. Across all species and treatments, *Daphnia* with species and body size‐specific large head/helmet were found to have also a species‐ and size‐specific large spina (*r* = .47, *p* < .05). Likewise, residual head size and residual ventral SBA (*r* = .49, *p* < .05), residual head size and residual ventral spinule length (*r* = .52, *p* < .05), and residual ventral SBA and residual ventral spinule length (*r* = .69, *p* < .0001) were significantly and positively related after correction for multiple testing. No co‐variation of trait expressions was observed between residual dorsal SBA and any other trait (Figure 4). Correlations conducted within species suggest that *D. galeata* had positive significant co‐variation between residual head size and spina size (*r* = .62, *p* < .05), between residual head size and residual ventral spinule length (*r* = .70, *p* < .05), between residual head size and residual ventral SBA (*r* = .70, *p* < .05), between residual spina size and residual ventral spinule length (*r* = .58, *p* < .05), and between residual ventral SBA and residual ventral spinule length (*r* = .71, *p* < .05). No significant co‐variation was found between traits of *D. longispina* and between residual head size and residual spina size of *D. cucullata*.

## 4 DISCUSSION

The invertebrate predators *Bythotrephes longimanus and Leptodora kindtii* are able to induce morphological defenses within all three species of the *D. longispina* complex. We found that the morphological responses of *D. longispina* species complex were trait‐, *Daphnia*—species, and predator—species‐specific. However, our results only partially support our expectations. *D. longispina* was indeed less responsive to the two predators as compared to the other two species but only regarding macrodefenses. Microdefenses were induced in *D. longispina* and *D. galeata*, but not in *D. cucullata*. Hence, microdefenses were expressed independently of macrodefenses across the three *Daphnia* species.

We did neither observe the induction of an expanded head form (helmet) nor spina elongation for *D. longispina*. The absence of helmet induction corresponds to the typical appearance of *D. longispina* in Lake Constance (Güde & Straile, 2016). This suggests that diel vertical migration of this species in Lake Constance seems to provide sufficient protection against the predation pressure of both invertebrate predators against the visual predator *Bythotrephes* but also against the tactile predator *Leptodora*. The lack of strong morphological responses here suggests a trade‐off may exist between behavioral and morphological defenses and suggests different strategies to deal with predation risk can evolve among species in this complex. The supposed behavioral protection seems to remove potential fitness benefits of elongated heads and spines, causing absence of helmets not only in situ, but also in our laboratory environment, in which daphnids were exposed to kairomones and light during daytime. Hence, helmet induction seems to be not a part of the defensive repertoire of *D. longispina* and is not only switched off in situ by DVM induced reduction of light availability. This is in contrast to life‐history antipredator strategies of *D. magna* in response to fish kairomones, which expression depends on light intensity (Effertz & von Elert, 2014).

Absence of *D. longispina* morphological defenses was also observed in Norwegian deep lakes (Sperfeld et al., 2020). However, *D. longispina* clones from small and shallow water bodies in Norway developed longer spines and neck teeth in response to *Chaoborus* kairomones (Sperfeld et al., 2020). Interestingly, some *D. longispina* with neck teeth have also been observed occasionally in Lake Constance (Güde & Straile, 2016) despite the absence of *Chaoborus* in this deep lake. As no neck teeth were observed in our experimental animals this might suggest that either a different predator induces neck teeth induction in Lake Constance *D. longispina*, or that neck teeth expression is highly clone‐specific. In the latter case, neck teeth were not part of the defensive repertoire of the five clones used in our study. This reasoning is consistent with the rarity of neck teeth observations in Lake Constance.

The strong response of *D. galeata* observed in this study confirms our recent study showing that *Bytotrephes* and *Leptodora* will induce helmets and longer spines in this species (Octorina et al., 2022). In the same study, using eight clones with replicates at the clonal level, the age at first reproduction was observed to be the latest and demographic costs, characterized by a reduced number of offspring, were found to be the highest in the presence of *Bythotrephes* treatment. Helmet formation of *D. galeata* is also typically observed in Lake Constance during the summer season (Güde & Straile, 2016). Likewise, helmet formation of *D. galeata‐mendotae* has been experimentally induced by other invertebrate predators such as *Chaoborus* and *Notonecta* (Dodson, 1988). However, *D. galeata* clones from various lakes, including Lake Constance, did not produce a helmet in response to fish kairomones (Tams et al., 2018). Longer spines in response to copepod kairomones have been observed for a *D. longispina* × *D. galeata* hybrid clone (Caramujo & Boavida, 2000), and for *D. galeata* and *D. galeata* hybrids in response to fish kairomones, however, the latter only at high food concentrations (Spaak & Boersma, 1997).

Induction of *D. cucullata* helmet formation in response to invertebrate predators is widespread and has been experimentally demonstrated in response to *Chaoborus*, copepod, and *Leptodora* kairomones (Laforsch & Tollrian, 2004). Likewise—and in contrast to our results—spina elongation was demonstrated in response to *Chaoborus* and *Leptodora* kairomones (Laforsch & Tollrian, 2004). Unfortunately, we lost two *D. cucullata* clones within the *Leptodora* treatment, and thus had a lower statistical power to detect effects of *Leptodora* kairomones for this species. We can therefore not exclude the possibility that also Lake Constance *D. cucullata* might elongate their spines in response to *Leptodora* and *Bythotrephes*.

Our study is the first to show the induction of three microdefenses in *D. longispina* and *D. galeata*, and the absence of these defenses in *D. cucullata*. Such induction of microdefenses has been reported previously in several *Daphnia* species, e.g., *D*. *barbata*, *D. similis*, *D*. *magna*, and *D*. *longicephala* (Herzog & Laforsch, 2013; Ritschar et al., 2020) in response to the invertebrate predators *Triops cancriformis* and *Notonecta maculata* suggesting that these defenses are widespread among *Daphnia*. However, no increase of dorsal and ventral SBAs, nor of spinule lengths of *D. magna* have been found in response to kairomones of a fish species, *Leucaspius delineatus*, suggesting that microstructure induction might also be a predator‐specific adaptation (Diel et al., 2021). Similarly, in *D. cucullata*, we found no dorsal or ventral spinules in any of the treatments, including the control and the two predator treatments. This suggest that also spinules are not expressed in all *Daphnia* species. However, we detected that spinules were present in neonates of *D. cucullata* in all treatments (data not shown), suggesting that the expression of microdefenses likely depends on developmental stage. Such stage‐specificity has been shown for neck teeth development of *D. pulex* in response to *Chaoborus* kairomones (Tollrian, 1995). However, the strongest evidence for microstructures as independent modules of the defense strategy and not merely by‐products of other defensive structures is the presence and length induction of microspines in *D. longispina* despite the absence of morphological macrodefenses.

The multitude of *Daphnia* induced defenses have been suggested to be uncoupled (Boersma et al., 1998) following a modular concept, which favors the evolution of defenses tailored toward individual predator species (Herzog & Laforsch, 2013). We found that body size‐adjusted traits in the *Daphnia longispina* complex co‐varied positively with other body size‐adjusted traits both between and within *Daphnia* species. For example, adjusted head, respectively, helmet sizes, were significantly related to all other traits besides dorsal SBA. In contrast, the expression of adjusted dorsal SBA was not related to any other morphological trait. This might suggest that specific combination of traits might be especially favorable in some predator environments and might act synergistically. In contrast, other traits might provide only additive benefit when exposed to predators, and thus show no co‐variation with other traits.

## 5 FUTURE RESEARCH

Within the world of plasticity research, one of the ideas that receives too little attention is how among trait relationships (trade‐offs or positive covariation) vary across environments and among species (Reger et al., 2018; Stearns, 1989). This idea that trade‐offs or positive co‐variation patterns are themselves plastic is important because if the traits involved relate strongly to fitness, as predator defense traits do, then context‐dependent variation in these relationships will be important to coexistence under variable environments.

While we cannot analyze our data to explore bivariate reaction norms across environments as proposed by Stearns (1989), we do have enough data to propose a compelling hypothesis that relationships between traits are predator‐environment dependent and that this dependency may vary among co‐occurring prey species that share two common predators.

**FIGURE 5:**
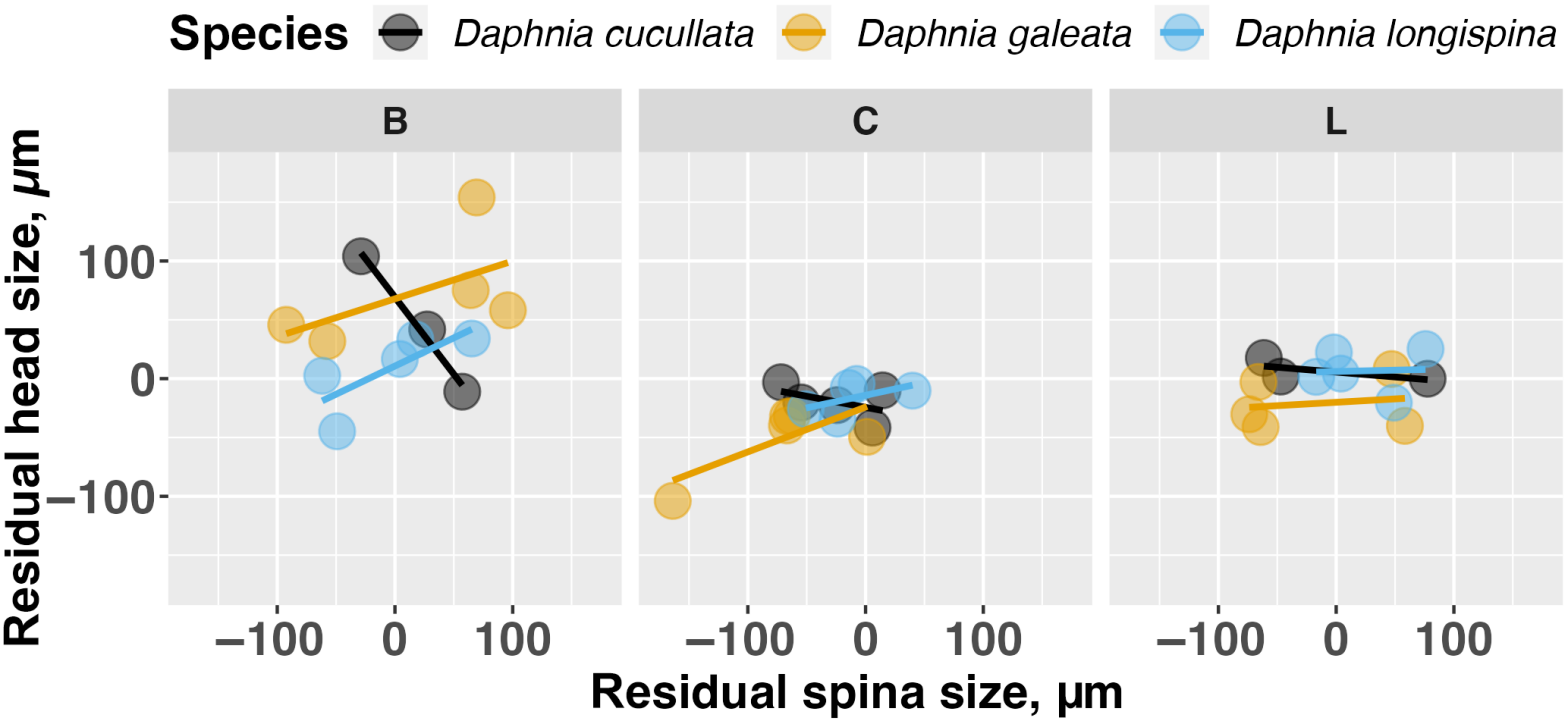
Relationship between residual head size and residual spina size among clonal means of each species in Lake Constance separately shown for the three predator treatments (*Bythotrephes* (B), Control (C), and *Leptodora* (L)). The lines represent the linear regressions of each species' residual head size as a function of its residual spina size.

For example, in Figure 5, we can see that patterns of G × E vary among species (the variation and sign of the relationship for each species change in different ways across the treatments). Variability in particular seems to manifest in different ways among species for each predator. For example, head size is more variable under *Bythotrephes* whereas spina size is more variable under *Leptodora*. One might argue that only with *Bythotrephes* as a predator, we see more variability in both residual head and spina sizes, possibly indicating a positive relationship for two species (*D. galeata* and *D. longispina*).

Of course these data, with only five genotypes, are too sparse to draw firm conclusions via statistics, but our core analyses highlight substantial genetic variation and across species variation suggesting that increasing the number of genotypes assayed can help reveal the context dependency of trait relationships. Such an effort could start to shed light on how trade‐offs and positive relationships among predator defense traits are tied to coexistence among multiple prey species sharing two predators.

This is the first study which explores induced defenses of *Daphnia* in a community context involving multiple *Daphnia* prey species, and multiple predators—all occurring in one lake ecosystem. Overall, our work demonstrates the presence of dorsal and ventral SBA microdefenses, highlighting the complexity of defensive mechanisms and their multifaceted contributions to survival strategies. Our study further reveals a captivating contrast between two *Daphnia* species. *D. cucullata* has a big helmet but no microdefenses. In contrast, *D. longispina* deploys microdefenses rather than macro‐morphological ones, which is consistent with modularity theory, but challenges the view that microdefenses are mere developmental by‐products of other induced morphological changes. The patterns in our data among three prey species and two predator species reveal a multifaceted set of strategies linking macro‐morphological characteristics and smaller‐scale defenses, which is further modified by the potential role of DVM behavioral responses in *D. longispina*. Clearly, further investigation is warranted to unveil the strategies defined across multiple types of induced traits among multiple species facing multiple predators.

## AUTHOR CONTRIBUTIONS

**Marjohn Yucada Baludo:** Conceptualization (equal); data curation (equal); formal analysis (equal); investigation (equal); methodology (equal); writing – original draft (lead); writing – review and editing (equal). **Pelita Octorina:** Data curation (equal); investigation (equal); methodology (equal); writing – review and editing (equal). **Andrew Beckerman:** Formal analysis (equal); writing – review and editing (equal). **Dietmar Straile:** Conceptualization (equal); formal analysis (equal); funding acquisition (lead); methodology (equal); project administration (lead); supervision (lead); writing – review and editing (equal).

## CONFLICT OF INTEREST STATEMENT

The authors declare no competing interests.

## Data Availability

The data generated and analyzed in this study are accessible through the research data repository of the University of Konstanz, KonDATA: https://doi.org/10.48606/108.
